# Fellowships, Grants, & Awards

**Published:** 2005-11

## The Environmental Fellows Program at Harvard University

The University Center for the Environment has created the Environmental Fellows program to enable recent doctorate recipients to use and expand Harvard’s extraordinary resources to tackle complex environmental problems. The Environmental Fellows will work for two years with Harvard faculty members in any school or department to create new knowledge while also strengthening connections across the university’s academic disciplines.

The fellowships will be awarded on a competitive basis. Candidates will propose a research program and secure a commitment from one or more Harvard faculty members to host the candidate’s work. Candidates should have received their terminal degree between May 2001 and September 2006. (Fellows must have completed all requirements of their degree before starting work in September 2006.) Candidates with a doctorate or equivalent in any field are eligible, and they may propose research projects in any discipline. Candidates who received terminal degrees from Harvard and postdocs currently working at Harvard are eligible for the fellowship, provided their research and host arrangements take them in new directions and forge new connections within the University.

The fellowship will provide an annual stipend of $50,000 plus health insurance, other benefits, and a $5,000 allowance for travel and professional expenses.

The Harvard University Center for the Environment expects to award up to eight fellowships in 2006 and an average of six per year thereafter. The center will organize a co-curricular program to ensure that the fellows get to know each other and each other’s work. All fellows are required to attend biweekly dinners with other fellows, faculty members, and guests.

The center encourages research and education about the environment and its many interactions with human society. The center draws its strength from faculty members and students across the university who make up a remarkable intellectual community of scholars, researchers, teachers, and practitioners of diverse fields. The center’s mission is to strengthen and expand that community by supporting research, encouraging faculty and students to apply their particular expertise to environmental topics, and providing a convivial space for collaboration. The center is located in the University’s Geological Museum at 24 Oxford Street, Cambridge.

Selection criteria: 1) Applicant’s prior success and potential contribution to scholarship or practice. 2) Project significance: the potential impact of the research project on scholarship at Harvard and on environmental problems. 3) Diversity: The committee will select a group of fellows from a range of academic disciplines whose work will focus on a variety of topics. Recipients and hosts may include people with degrees in the sciences, economics, law, government, public policy, public health, medicine, design, and the full array of humanities. Their research topics will be equally varied. Interdisciplinary research projects are encouraged, although this is not a requirement for the fellowship. Candidates with interests in a single discipline are encouraged to apply. 4) Host’s commitment: the host faculty member’s enthusiasm for the proposed project, his or her ability to mentor the fellow, and his or her ability to provide office space and a productive work environment.

Potential candidates should start early to identify and establish a relationship with a Harvard faculty member to host his or her research. The host will be a mentor to the fellow and will provide office space and basic administrative support. The host may not be the candidate’s thesis adviser. The host must, however, submit a letter of support (maximum of two pages) to the selection committee describing in detail the level of commitment to the research and the candidate.

Applicants unfamiliar with Harvard faculty members will find many of them listed on the center’s web pages organized both by academic areas (economics, engineering) and by research topics (climate, human health). Most faculty members have their own web pages which will provide much more detailed information about publications and interests. Applicants are encouraged to use the center’s faculty lists as a starting point. Any faculty member from any discipline can serve as a host, regardless of whether the host has had prior experience with environmental research.

Applicants, referees, and hosts may e-mail all portions of the application to the center, attaching all documents to the e-mail as PDFs or Word files. Referees and hosts should e-mail or mail their letters directly to the center.

A complete application includes 1) a cover sheet (see below); 2) a detailed research proposal (a maximum of 5 pages, including illustrations; 12-point type); 3) a letter of support from the applicant’s host committing to serve as a mentor and explaining his or her commitment to the proposed research, including the provision of office space and any financial commitments; 4) curriculum vitae including list of publications; 5) letters of reference from at least three professional colleagues, including the applicant’s dissertation adviser.

Submit applications and letters of reference by e-mail to the Harvard University Center for the Environment by January 15, 2006. The center will announce its selections by March 1, 2006. Fellows must start work the following September. For information about application requirements contact Richard A. Minard, Jr., Harvard University Center for the Environment, 24 Oxford Street, 3rd Floor, Cambridge, MA 02138 USA, 617-495-0368, e-mail: Richard_Minard@harvard.edu.

## Established Investigator Award in Cancer Prevention & Control

The objective of the NCI Established Investigator Award in Cancer Prevention and Control (K05) is to provide qualified researchers with protected time to devote to research and mentoring. The award is designed for established scientists who have already demonstrated a sustained, high level of research and mentoring productivity and who need K05 support to continue these activities. The award provides partial salary support for up to 5 years and for up to 50 percent effort. It is renewable for one additional 5-year period. Examples of cancer prevention and control research and mentoring activities supported by this funding opportunity include, but are not limited to, the following areas: 1) the identification of modifiable risk factors for cancer, such as nutrient intake, exercise, exposure to carcinogens, or behavioral lifestyle factors; 2) molecular epidemiology, to identify allelic variants in genes in relation to cancer incidence or course; 3) studies of interactions of genetic and endogenous factors (e.g., hormonal milieu) with exogenous risk factors as related to cancer incidence and course; 4) the identification of community structural and social variables (e.g., location of health care facilities, access to health care, culturally conditioned attitudes affecting health behaviors) that are barriers to or facilitators of cancer prevention and control efforts; 5) studies of the above factors in relation to health disparities in cancer incidence and outcomes; 6) identification of biomarkers and clinical/screening studies of their utility as predictors of cancer risk and outcome; 7) behavioral research to identify cognitive or motivational attributes that affect the individual's acceptance of screening guidelines or treatments and propensity to engage in health-promoting or cancer risk-reducing behaviors; 8) the development of preventive interventions to decrease cancer risk behaviors and/or increase health-promoting behaviors; 9) chemoprevention from studies of the identification and early-phase characterization of candidate agents to preventive intervention trials; 10) studies of nutritional supplements or complementary/alternative interventions in relation to cancer prevention and control; 11) health services research, including patient outcome studies, practice research, and medical decision analyses related to cancer prevention or to cancer care and patient outcomes; 12) palliative care studies, including interventions to improve quality of life; 13) survivorship research, including studies of outcomes and quality of life as related to cancer course, treatments, and treatment side effects; and 14) studies of the effectiveness of cancer health communications in reducing high risk behaviors or in increasing participation in screening activities.

This funding opportunity will use the K05 award mechanism. The Established Investigator Award in Cancer Prevention & Control is a special NCI modification of the NIH Senior Scientist Award or K05 grant mechanism. In addition, the institution must demonstrate a commitment to the candidate and the candidate's goals for research, career development, and mentoring.

This funding opportunity uses the just-in-time budget concepts. It also uses the nonmodular budget format described in the PHS 398 application instructions (see http://grants.nih.gov/grants/funding/phs398/phs398.html). The applicant should follow the PHS 398 instructions for budget information, providing only the total direct costs requested for each year and for the entire proposed period of support, and provide budget justification information. For further assistance contact GrantsInfo, 301-435-0714, (telecommunications for the hearing impaired: TTY 301-451-0088) or by e-mail: GrantsInfo@nih.gov.

Applications must be prepared using the most current PHS 398 research grant application instructions and forms. Applications must have a Dun & Bradstreet (D&B) Data Universal Numbering System number as the universal identifier when applying for Federal grants or cooperative agreements. The D&B number can be obtained by calling 866-705-5711 or through the web site at http://www.dnb.com/us/. The D&B number should be entered on line 11 of the face page of the PHS 398 form.

The deadline for receipt of applications is July 2, 2008. The full PA is available at http://grants.nih.gov/grants/guide/pa-files/PAR-05-145.html

Contact: Mary C. Blehar, Cancer Training Branch, National Cancer Institute, 6116 Executive Boulevard, Suite 7019, MSC 8346, Bethesda, MD 20892-8346 USA, 301-496-8580, fax: 301-402-4472, e-mail: mblehar@mail.nih.gov.

Reference PAR-03-149

## Fall 2006 Greater Research Opportunities (GRO) Undergraduate Student Fellowships

The U.S. Environmental Protection Agency (EPA), as part of its Greater Research Opportunities (GRO) program, is offering Undergraduate Fellowships for bachelor level students in environmentally related fields of study. The deadline for receipt of preapplications is November 3, 2005. Subject to availability of funding, the agency plans to award approximately 15 new fellowships by July 21, 2006. Eligible students will receive support for their junior and senior years of undergraduate study and for an internship at an EPA facility during the summer between their junior and senior years. The fellowship provides up to $17,000 per year of academic support and up to $7,500 of internship support for a three-month summer period.

The GRO Undergraduate Fellowship program, like its predecessor (the Minority Academic Institution or MAI program), is intended to strengthen the environmental research capacity of institutions of higher education that receive limited funding to build such capacity, including in particular institutions with substantial minority enrollment. The program supports quality environmental education for undergraduate students, thereby encouraging them to pursue careers in environmentally related fields and to continue their education beyond the baccalaureate level. This goal is consistent with the mission of EPA, which is to provide leadership in the nation’s environmental science, research, education, assessment, restoration, and preservation efforts. This program will benefit both the public and private sectors which will need a steady stream of well-trained and diverse environmental specialists if our society is to meet the environmental challenges of the future.

It is anticipated that a total of approximately $622,500 will be awarded under this announcement, depending on the availability of funds. The EPA anticipates funding approximately 15 fellowships under this RFA. The projected award per fellowship is $17,000 per year total costs, for up to 2 years. The EPA reserves the right to reject all preapplications and make no awards under this RFA. The EPA reserves the right to make additional awards under this RFA if additional funding becomes available. Any additional selections for awards will be made no later than 4 months after the original selection.

You may submit either a paper or an electronic preapplication (but not both) for this announcement. For paper preapplications, forms can be found on the NCER web site: http://es.epa.gov/ncer/rfa/forms/. For electronic preapplications, use the preapplication package available at https://apply.grants.gov/forms_ apps_idx.html (see "Submission Instructions for Electronic Pre-Applications").

Contact: Georgette Boddie, 202-343-9741, e-mail: boddie.georgette@epa.gov.

## The Obese and Diabetic Intrauterine Environment: Long-Term Metabolic or Cardiovascular Consequences in the Offspring

It has long been recognized that the intrauterine environment can have profound effects on the development and health of the fetus. Alterations in maternal nutritional status resulting from caloric or protein restrictions as well as specific micronutrient deficiencies have been shown to impact fetal development. Maternal undernutrition can lead to intrauterine growth retardation and has been shown to increase the risk of metabolic disorders such as diabetes, hypertension, and cardiovascular disease in the offspring.

Emerging evidence suggests that maternal over-nutrition may have similar long-term metabolic consequences in the offspring as those seen with undernutrition. Recent animal studies suggest that the pups of obese dams develop obesity and gain more weight than offspring of normal-weight rats. In rodents, maternal high-fat or cholesterol over-feeding during pregnancy results in offspring with elevated risk factors for cardiovascular disease such as increased blood pressure, abnormal lipid profiles and abnormal glucose homeostasis. Fat-rich diets have also been shown to produce endothelial dysfunction in the offspring. In humans, the reported increases in prepregnancy body mass index and increased weight gain during pregnancy, particularly in obese women, are associated with increases in neonatal weight and body adiposity. Furthermore, several studies have shown that fetuses and children born of hypercholesterolemic mothers have an increased incidence of fatty streaks in the aorta. Recent studies in the Pima Indian Population indicate that intrauterine exposure to diabetes significantly increases systolic blood pressure (SBP) and hemoglobin A1c (HbA1c) during childhood. Evidence also exists that increased birth weight is positively correlated with subsequent risk of cancer. As infant and childhood obesity can predict obesity in the adult, these data suggest that maternal obesity may exacerbate an already alarming incidence of obesity and potentially Type II diabetes in the general population. Understanding the mechanisms by which the obese and diabetic maternal intrauterine environment elicits permanent metabolic and cardiovascular disease in the fetus will provide a basis for future interventional studies in humans.

Maternal insulin resistance as observed in obese women and women with gestational diabetes has been associated with increased fetal fat mass. Leptin, elevated in obesity, has been shown to alter placental gene transcription and cell proliferation and it is possible that other cytokines, also increased in obesity, may play a role as well. The adipose tissue also secretes factors that are implicated in inflammatory processes, blood pressure, coagulation and fibrinolysis that can influence the development of cardiovascular disease. In addition, islet cell or adipocyte development and morphology may be altered. However, the exact mechanisms by which maternal obesity increases the risk of metabolic and cardiovascular disease in the offspring have not been established. Potentially permanent changes may be occurring at the genetic, cellular, or tissue level, either peripherally or at the level of the central nervous system.

Neural pathways regulating food intake, body weight, and the cardiovascular system may be particularly vulnerable to the metabolic status of the obese or diabetic mother. Leptin, through direct central effects, can affect the sympathetic nervous system and lead to hypertension and/or heart disorders. Recent studies demonstrate that leptin exerts a trophic effect on hypothalamic neurons and that the pathways involved in feeding regulation exhibit extensive neuroplasticity in response to metabolic perturbations, even during the postnatal period. However, the role of postnatal nutrition in the development of metabolic, cardiovascular disease, or cancer in the offspring of obese mothers is not clear. Maternal under-nutrition in both animal and population-based studies indicate that the detrimental effects are primarily manifested when postnatal nutrition is excessive relative to the intrauterine environment but therapeutic windows with respect to the consequences of maternal obesity have not been identified or defined.

The objective of this RFA is to support mechanistic research investigating the effects of maternal obesity, gestational diabetes, or diabetes on the development of obesity, and other metabolic and cardiovascular diseases, or cancer in the offspring. This is a new and burgeoning area with important clinical implications. Thus, the applications solicited by this RFA should not only elucidate factors involved in the etiology of obesity in the offspring of obese mothers but in addition, should provide the scientific basis whereby future prevention and intervention studies in humans can be developed. Proposed studies in murine, rat, and large animal models, like sheep and nonhuman primates, should be focused on identifying the potential mechanisms mediating the proposed long-term consequences of maternal obesity or diabetes on the offspring. Outcome variables should not be limited solely to the weight of the offspring but should also include specific measurement of biological endpoints in appropriate tissues such as the brain, adipocyte, heart, and vasculature, placenta, endothelium, mammary gland or pancreatic islet. Pilot studies demonstrating the feasibility of conducting ethical and appropriate studies in humans will also be considered. Studies conducted in nonhuman primates and where possible, in humans, are encouraged.

Potential research topics include, but are not limited to: 1) Defining critical periods of plasticity and/or susceptibility to metabolic perturbations in the maternal environment for neural pathways involved in the regulation of food intake, motivation, body adiposity, and the cardiovascular system in the rodent and non-human primates. 2) Development of appropriate animal models to facilitate determination of the relative roles of the genetic, maternal *in utero* environment, and postnatal environment. 3) Brain imaging studies to study development of neural pathways involved in regulation of food intake and motivational pathways associated with food intake in humans and nonhuman primates. 4) Imaging of the adipocyte, heart and vasculature, placenta, pancreatic b-cell, body adiposity, or body composition of the offspring to determine the effects of maternal obesity and diabetes. 5) Mechanistic investigations of the role of stress at a cellular or systemic level as a mediator of the long-term consequences of maternal obesity and diabetes. 6) Mechanistic investigations on the effect of maternal obesity and diabetes on factors that influence the development of cardiovascular disease in the offspring. Examples include lipid metabolism, inflammation, vascular reactivity, hypertension, endothelial dysfunction, endocrine systems, and hormones. 7) Mechanistic investigations on the effect of maternal obesity and diabetes on factors that influence the development of cancer in the offspring. 8) Investigation of the role of epigenetics as a mechanism mediating the effects of maternal obesity and diabetes on development of metabolic, cancer, or cardiovascular disease in the fetus.

This funding opportunity will use the R01 and R21 award mechanisms. This funding opportunity uses just-in-time concepts. It also uses the modular as well as the nonmodular budget formats (see http://grants.nih.gov/grants/funding/modular/modular.htm). Specifically, if you are submitting an application with direct costs in each year of $250,000 or less, use the modular budget format described in the PHS 398 application instructions, available at http://grants.nih.gov/grants/funding/phs398/phs398.html. Otherwise, follow the instructions for nonmodular research grant applications. For further assistance contact GrantsInfo, 301-435-0714 (telecommunications for the hearing impaired: TTY 301-451-0088) or by e-mail: GrantsInfo@nih.gov.

Applications must be prepared using the most current PHS 398 research grant application instructions and forms. Applications must have a D&B Data Universal Numbering System (DUNS) number as the universal identifier when applying for federal grants or cooperative agreements. The D&B number can be obtained by calling 866-705-5711 or through the web site at http://www.dnb.com/us/. The D&B number should be entered on line 11 of the face page of the PHS 398 form.

The deadline for receipt of letters of intent is February 16, 2006, with March 16, 2006 the deadline for receipt of applications. The complete version of this RFA is available at http://grants.nih.gov/grants/guide/rfa-files/RFA-DK-05-014.html.

Contact: Karen Teff, Division of Diabetes, Endocrinology and Metabolic Diseases, National Institute of Diabetes, Digestive and Kidney Disease Democracy II, Room 734, 6707 Democracy Boulevard, Bethesda, MD 20892 USA, 301-451-7335, fax: 301-480-0475, e-mail: kt216q@.nih.gov; Cristina Rabadan-Diehl, Division of Heart and Vascular Diseases, National Heart, Lung, and Blood Institute, Rockledge II, Room 10186, 6701 Rockledge Drive, Bethesda, MD 20892 USA, 301-435-0550, fax: 301-480-2858, e-mail: cr225k@nih.gov; Cindy Davis, Division of Cancer Prevention, National Cancer Institute, 6130 Executive Boulevard, EPN Room 3159, Bethesda, MD 20892 USA, Rockville, MD 20852 USA (express/courier service), 301-594-9692, fax: 301-480-3925, e-mail: davisci@mail.nih.gov. Reference RFA-DK-05-014.

